# Terms of debate: Consensus definitions to guide the scientific discourse on visual distraction

**DOI:** 10.3758/s13414-023-02820-3

**Published:** 2024-01-04

**Authors:** Heinrich R. Liesefeld, Dominique Lamy, Nicholas Gaspelin, Joy J. Geng, Dirk Kerzel, Jeffrey D. Schall, Harriet A. Allen, Brian A. Anderson, Sage Boettcher, Niko A. Busch, Nancy B. Carlisle, Hans Colonius, Dejan Draschkow, Howard Egeth, Andrew B. Leber, Hermann J. Müller, Jan Philipp Röer, Anna Schubö, Heleen A. Slagter, Jan Theeuwes, Jeremy Wolfe

**Affiliations:** 1 Department of Psychology, University of Bremen, Hochschulring 18, D-28359 Bremen, Germany; 2 The School of Psychology Sciences and The Sagol School of Neuroscience, Tel Aviv University, Ramat Aviv 69978, POB 39040, Tel Aviv, Israel; 3 University of Missouri, Columbia, MO USA; 4 University of California Davis, Daivs, CA USA; 5 University of Geneva, Geneva, Switzerland; 6 York University, Toronto, ON Canada; 7 University of Nottingham, Nottingham, UK; 8 Texas A&M University, College Station, TX USA; 9 University of Oxford, Oxford, UK; 10 University of Münster, Münster, Germany; 11 Lehigh University, Bethlehem, PA USA; 12 Carl von Ossietzky Universität Oldenburg, Oldenburg, Germany; 13 Johns Hopkins University, Baltimore, MD USA; 14 The Ohio State University, Columbus, OH USA; 15 Ludwig-Maximilians-Universität München, München, Germany; 16 Witten/Herdecke University, Witten, Germany; 17 Philipps University Marburg, Marburg, Germany; 18 Vrije Universiteit, Amsterdam, The Netherlands; 19 Harvard Medical School, Boston, MA USA; 20 Brigham & Women’s Hospital, Boston, MA USA

**Keywords:** Visual search, Visual distraction, Taxonomy, Definitions, Adversarial collaboration

## Abstract

Hypothesis-driven research rests on clearly articulated scientific theories. The building blocks for communicating these theories are scientific terms. Obviously, communication – and thus, scientific progress – is hampered if the meaning of these terms varies idiosyncratically across (sub)fields and even across individual researchers within the same subfield. We have formed an international group of experts representing various theoretical stances with the goal to homogenize the use of the terms that are most relevant to fundamental research on *visual distraction* in visual search. Our discussions revealed striking heterogeneity and we had to invest much time and effort to increase our mutual understanding of each other’s use of central terms, which turned out to be strongly related to our respective theoretical positions. We present the outcomes of these discussions in a glossary and provide some context in several essays. Specifically, we explicate how central terms are used in the distraction literature and consensually sharpen their definitions in order to enable communication across theoretical standpoints. Where applicable, we also explain how the respective constructs can be measured. We believe that this novel type of adversarial collaboration can serve as a model for other fields of psychological research that strive to build a solid groundwork for theorizing and communicating by establishing a common language. For the field of visual distraction, the present paper should facilitate communication across theoretical standpoints and may serve as an introduction and reference text for newcomers.

## Introduction

Science travels on its vocabulary. Scientific progress is impeded if words are not used consistently and effectively. It appears trivial to note that for any reasonable debate, the debaters must agree on the meaning of the words they use for debating. Yet, a closer look reveals the striking absence of consensus regarding the meaning of even the most central terms in many areas of psychology. This state of affairs inevitably produces misunderstandings and quashes hope for theoretical consensus.

Consider this historical example: Only when “heat” was distinguished from “temperature” did the science of thermodynamics proceed (Roller, 1950). The terms *heat* and *temperature* describe distinct but obviously related attributes of matter. Before the 19th century, heat was regarded as a liquid called "caloric" that flowed from the hotter to the colder object. Not until the 1760s was a distinction made between the quantity (caloric) and the intensity (temperature) of heat. Another 100 years passed before the misguided idea of flowing caloric was abandoned. Today, we understand that heat corresponds to the total energy within a mass: a kettle of boiling water has more heat than a thimble of boiling water. In contrast, temperature measures the average kinetic energy per molecular or atomic constituent: the temperature of boiling water is 100 °C in both the kettle and the thimble.

The widespread practice of ascribing technical meanings to common words creates a particular challenge for research in the cognitive sciences. This literature is full of words like “attention” and “distraction.” When a psychologist writes terms like these, she does not necessarily mean the same thing as a lawyer or the person in the street. Her usage of these terms is highly specific, because sharp and distinct definitions are necessary in order to operationalize these constructs and uncover their underlying mechanisms. Moreover, such specificity allows communicating scientific ideas efficiently among experts. But what if even scientists from related fields use these terms differently, and worse still, they are unaware of this fact? In that case, the psychologist’s findings will most probably be misconstrued by, say, a neurophysiologist; only if the psychologist and neurophysiologist mean the same thing when they speak about attention and distraction will the psychologist's work be relevant for the neurophysiologist. The same holds for communication across the various subfields of psychology and many additional neighboring disciplines, such as computer science, philosophy, economics, and pedagogy. Enabling communication within and across disciplines is what we aim for, and this is why we have invested much effort to define the terms used in our research community. Our goal is to replace habitual, unprincipled use of terms with more disciplined usage. Such discipline can, in a later step, be organized by formal mathematical models that convert abstract terms into concrete assumptions and specific equations.

Our objective here is to clearly define how terms are used by the research community investigating *visual distraction* from a fundamental-research perspective, which mainly consists of cognitive psychologists (for taxonomies pertaining to attention, see also Chun et al., 2011; Hommel et al., 2019; Kaldas, 2022). For some terms, providing definitions that would be more broadly accepted, that is, beyond our field, turned out to be impossible. Here are two examples that illustrate why this is the case. We use the word *paradigm* to describe types of experimental setups (e.g., *spatial cueing paradigm*), yet in philosophy of science, the term is more commonly used to refer much more broadly to a set of assumptions within which science is performed at any given time in history – with *paradigm shift* referring to a disruptive change in these assumptions (Kuhn, 1962). Likewise, our research community typically uses the word *stimulus* to refer to a single item within a search array in a typical visual-search task, whereas outside this community, *stimulus* often refers to everything that is presented to the senses at a given point in time (i.e., the whole search array). Despite such idiosyncratic use of certain terms in our research community, our endeavor will be useful for interdisciplinary exchange, because clear definitions will allow scientists from neighboring disciplines as well as applied researchers and practitioners interested in distraction (e.g., Dontre, 2021; Overton et al., 2015) to look up what a term means in the fundamental-research community on visual distraction.

This exercise is important beyond issues of relevance outside the laboratory and communication across disciplines. In fact, while working on this paper, it turned out that even within the small and friendly fundamental-research community investigating visual distraction, many terms have been used differently by different authors. It thus became clear that finding definitions of these words that we could all agree upon would be of great help for conceiving more discriminating hypotheses and designing more informative experiments. Originally, we feared that this linguistic treatment might appear as dry as bones and bore most readers interested in understanding the mechanisms of attention and distraction. Still, we argued, if the skeleton is weak or malformed, on what can the flesh of the mechanism hang and move? The present paper was mainly an attempt to provide this skeleton for fundamental research on *visual distraction*. In hindsight, however, we feel that the end product has much more to offer. Apart from being a reference text to understand or double-check the meanings of central terms, our paper provides a particularly accessible entry point into the field for novices: it explains central theoretical concepts, outlines measurement methods and their common interpretations, and provides a condensed overview of current theoretical stances, as well as pointers to the relevant literature. For experts (including ourselves), this paper will serve to more clearly distinguish between mere linguistic misunderstandings and actual theoretical discrepancies and therefore inform future empirical and theoretical work to be more focused on the latter.

Despite its potential value for facilitating scientific communication, our endeavor is not free from danger. As a reviewer aptly explained, “the danger is of calcification, of the reinforcement of preconceptions and status quo.”^1^ In particular, the definitions of terms – or even their mere existence – might put constraints on the phenomena that can be examined. For example, accepting that search displays contain stimuli that have certain features renders it difficult to discuss search situations in which what *search display*, *stimulus*, and *feature* refer to is less clear: for instance, when your friend asks you to pick up their car keys from their home, what counts as the search display? What among the messy and dynamic visual stimulation can be considered a stimulus? If you are not familiar with their keys, you might not even be looking for a specific *feature* as defined here, because our definition includes mainly sensory aspects, such as hue and orientation, and does not encompass “car-keyness”. Thus, with increasingly clear and established definitions, the examined phenomena might become more and more abstract, and move us further away from the real-world phenomena we aim to understand. Despite this potential danger, we believe that the present manuscript provides a reference point from which it will be easier to develop definitions applicable to search situations in which some of the definitions laid out here are not useful.^2^.

It is important to keep in mind that some of the definitions provided here are tied to the current state of knowledge in our field. As new data accumulate, some of these might have to be updated, and new terms might have to be introduced. The main thrust of our endeavor, however, is that unnecessary proliferation of terms, as well as ambiguity and imprecision with regard to their definition seriously hamper scientific progress. We hope that promoting a more rigorous attitude towards terminology will discourage authors from obfuscating theoretical concepts by employing unspecific or misleading terms to immunize their own theory against criticism.

### Outline and reading suggestions

We are concerned with visual distraction, usually in situations in which multiple stimuli are present and the task is to find one particular target stimulus. This general paradigm, termed *visual search*, can be considered a model for selecting information in static visual scenes. The outcome of our endeavor and the main body of this paper describe various aspects of this paradigm, its theoretical underpinnings, and how it is used to study visual distraction. In a glossary complemented by a series of essays, we provide definitions of central terms. This treatment of terminology on visual distraction does not attempt to be complete: it focuses on those terms that appeared most central to our group of experts on in their theorizing on the topic and so excludes additional terms used in this literature. In addition to conceptual definitions, we also provide operational definitions. That is, in addition to clarifying what a term means, we also explain how the respective theoretical construct has been or could be measured or manipulated. In some cases, different words are used for the same construct, and we provide a list of synonyms.

The paper is divided into three sections: Part 1 (essays), Part 2 (glossary), and Appendix (genesis). Part 1 consists of several essays on what we refer to as *clusters of terms*, that is, terms that are strongly related and often obtain their meaning in relation to each other (see Table 1 for a list of these clusters). Each essay also provides pointers to the relevant literature. Terms that are defined in a given essay are printed in bold and cross-references to other essays are indicated by an arrow (→ ***Cluster X***).

**Table 1:** Clusters (essays) and the terms they cover

Clusters	Terms
Stimuli	Stimulus, set size, target, irrelevant stimuli, nontargets, distractors, target-matching distractor, salient distractor, singleton distractor, nonsingleton distractor informative/non-informative cue
Features	Target-defining feature, search-guiding feature, pre-attentive feature, reported feature, relative/absolute feature, first-order/second order feature, salience, singleton
Tasks	Task, detection task, localization task, discrimination task, search performance
Paradigms	Paradigm, additional-singleton paradigm, distractor interference, split-block paradigm, dot-probe paradigm, probe-detection performance, letter-probe paradigm, spatial-cueing paradigm, contingent-capture paradigm, cue-validity effect, attentional-blink paradigm, irrelevant-singleton paradigm, distractor-cueing paradigm
Templates and strategies	Target template, negative template, attentional control setting, attentional strategy, singleton-detection mode, feature-search mode, optimal tuning of attention, relational coding, dimension weighting
Types of distraction	Distraction, attentional capture, filtering costs, distractor interference, response-compatibility effects
Priority map	Priority map, salience map, conspicuity map, dimensional map, overall (salience or priority) map
Guidance	Guidance, stimulus-driven, bottom-up, top-down, goal-directed, selection history, feature/space/time-based attentional control, statistical learning, value learning, intertrial priming, scene guidance
Modulation	Inhibition, suppression, attentional suppression, attentional enhancement, enhancement effect, suppression effect
Timing	Proactive, reactive, temporal expectations, temporal structure

Part 2 is a glossary containing the most central terms, presented in alphabetical order. It can be used to quickly retrieve the definition of a specific term or serve as an entry point to the more extensive treatment of the terms in the corresponding essay (indicated by → ***Cluster X***, at the end of the definition), which provides context to the definition. This glossary is of core relevance, because the definitions it provides were most intensively discussed in the group and therefore constitute the closest we could get to an agreement. In case of discrepancies between essays and glossary definitions, the latter therefore take precedence. Note that not all the terms defined in the essays have their own entry in the glossary.

We foresee that this manuscript will not only be useful for scholars interested in visual distraction and related phenomena but might also provide a template for similar endeavors in other research communities. Therefore, the Appendix is a brief account of how the present paper developed, which might serve as a source of inspiration for similar future projects in other research communities.

### Part 1: Essays

#### Stimuli

##### Terms

Stimulus, set size, target, irrelevant stimuli, nontargets, distractors, target-matching distractor, salient distractor, singleton distractor, nonsingleton distractor, informative/non-informative cue

**Fig. 1:**
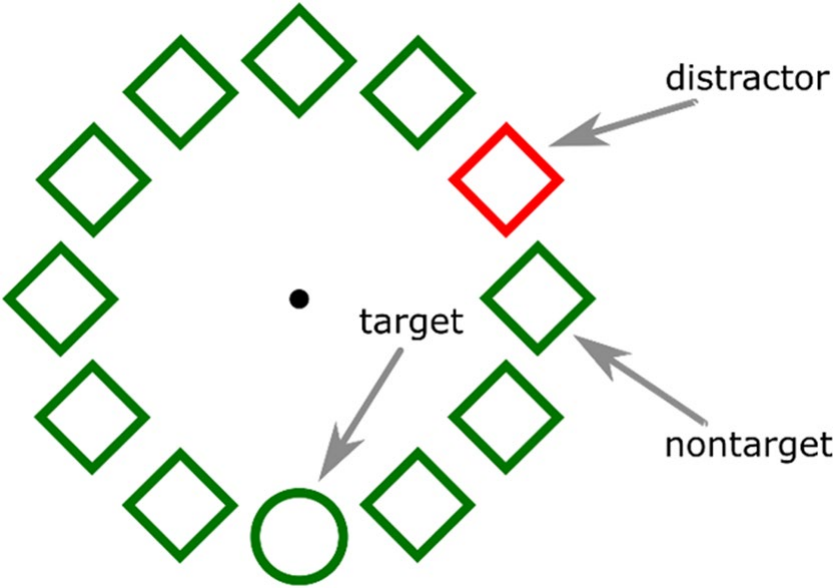
A visual-search display with set size 12 (i.e., consisting of 12 stimuli). The shape oddball (the circle) is the target and the color oddball (the red diamond) is a salient distractor. The green diamonds are nontargets or, in other contexts, distractors (see main text)

A visual-search display consists of at least one **stimulus**, but usually multiple *stimuli*, also sometimes referred to as *items* or *objects* (see Fig. 1 for an example). The number of stimuli within a search display is often manipulated in visual-search studies and referred to as **set size** (or *display size*). The stimulus that observers are instructed to find within a display is called a **target**. Although in many cases there is a single target that can be either present or absent, in some paradigms multiple targets can be simultaneously present. Instructions define the targets by one or more of their features (“look for the circle”), by their category identity (“look for a person”), by stating in which feature dimension they stand out (“look for the shape oddball”), or by stating that they stand out in any dimension (“look for any oddball”; → ***Features***).

Apart from the target(s), all other stimuli in the display are **irrelevant stimuli**, in that their processing does not contribute to achieving the task goals and in that *search performance* (→ ***Tasks***) would be better if the irrelevant stimuli were not processed. For certain types of searches, referred to as *inefficient* or *serial search*, processing multiple irrelevant stimuli in some detail can be difficult to avoid. An irrelevant stimulus might share some features with the target within or across trials (e.g., color in Fig. 1), but it never conforms to the definition of the target as given in the task instructions; that is, participants must be able to tell it apart from the target.

In the broader visual-search literature, all irrelevant stimuli are relatively interchangeably referred to as **nontargets** or **distractors**. In the literature on visual distraction, the typically unique stimulus for which the potential to attract attention is examined is most often referred to as a *distractor*, and more rarely as a *lure* or a *foil*. It might have this potential for instance because it (partially) matches the target definition or because it is salient (→ ***Features***). We recommend specifying what type of distractor is meant by adding an attribute such as **target-matching distractor** or **salient distractor** in order to clearly demarcate it from other irrelevant stimuli in the respective paradigm. Some researchers also stress the distinction between **singleton** and **nonsingleton distractors** (→ ***Features;*** → ***Suppression***).

The word *nontarget* could in some paradigms, in particular those inducing *pop-out* or *parallel search*, be reserved for those irrelevant stimuli that are not particularly likely to attract attention. When nontargets are highly homogeneous and densely packed (e.g., Fig 1) they mainly constitute the background. Thus, depending on the *attentional strategy* (→ ***Templates and strategies***) the same stimulus might act as a distractor or as a nontarget in this sense: for example, when searching for a red vertical target among green vertical and red horizontal irrelevant stimuli, that is, when performing a *conjunction search*, the red horizontal stimuli are distractors when the strategy is to focus on red stimuli and they are nontargets when the strategy is to focus on vertical stimuli.

In the *spatial-cueing paradigm* (→ ***Paradigms***), an irrelevant stimulus presented before search-display onset is often referred to as a **cue**. Such a (spatially) **non-informative cue** should not be confused with an **informative cue**, which provides advance information on the upcoming search display. Note that a non-informative cue in the context of the spatial cueing paradigm is sometimes referred as “the distractor”.

#### Features

##### Terms

Target-defining feature, search-guiding feature, pre-attentive feature, reported feature, task-irrelevant feature, relative/absolute feature, first-order/second order feature, salience, singleton

Visual stimuli can be described as a combination of features (also referred to as *properties* or *attributes*). These can range from the relatively simple, like color, to the complex, like whatever feature or combination of features allows people to recognize a stimulus as a “cat.” The features of a stimulus can play various roles in the context of a visual-search task. Obviously, there must be **target-defining features** that determine whether or not a particular stimulus is the search target. In instructions, the target-defining features might be simple and precise (e.g., find the red stimulus or the letter "T") or complex and difficult to describe as any collection of specific features (e.g., find any animal or determine whether there are clinically significant findings in this x-ray).

A second class of features are **search-guiding features** that can be used to direct attention to potential targets and/or away from stimuli that are not targets, but might also misguide attention (→ ***Types of distraction***). These features are often labeled **pre-attentive features,** based on the claim that they guide attention to a stimulus that has not yet been selected for attentional processing. While a target-defining feature can be essentially any visual property, the set of search-guiding features is limited. There are a few, probably up to two dozen, such search-guiding feature dimensions. It is largely agreed that color can guide attention, and that presence versus absence of an intersection, for instance, cannot. However, there is less consensus with regard to many other dimensions. For an extensive summary of potential search-guiding feature dimensions, see Wolfe and Horowitz (2017).

**Fig. 2:**
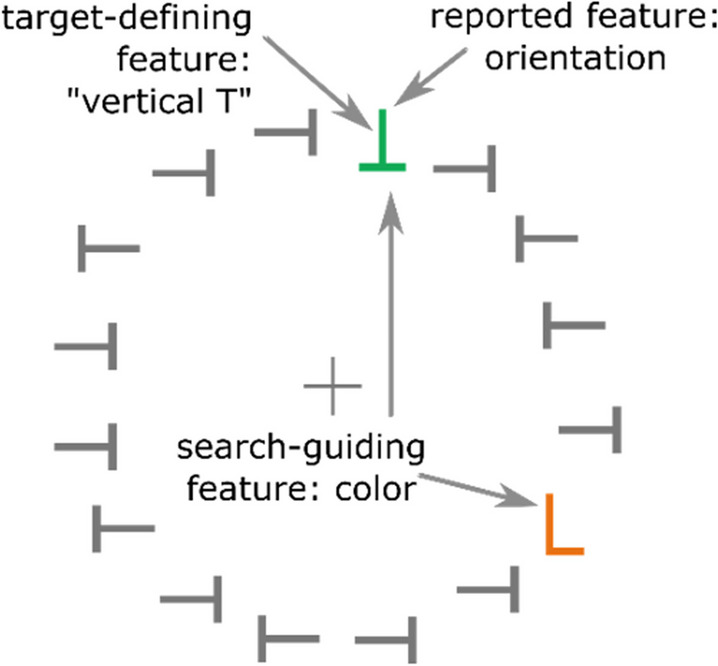
Illustration of different types of features when the task is to identify the orientation of a colored vertical T (adapted from Hilimire et al., 2010, Exp. 2)

For an example where search-guiding and target-defining feature differ, consider Experiment 2 of Hilimire et al. (2010; Fig. 2). Here, the search-guiding feature is "colored" and the target-defining feature is "vertical T " (for other examples, see Liesefeld et al., 2017, 2022; Woodman & Luck, 1999). While some tasks only require observers to decide whether or not a certain target is present (*detection task* → ***Tasks***), the task in Fig. 2 adds a **reported feature** or *response-defining feature*: the observer is asked to report the orientation of the T (*discrimination task* → ***Tasks***). Note that the term “response feature” is sometimes used but this can be ambiguous, as it might also refer to features of the motor response (e.g., the right index finger was used to press a button; Frings et al., 2020). **Task-irrelevant features** are those that are not target defining, not (potentially) search guiding, and not reported.

**Fig. 3:**
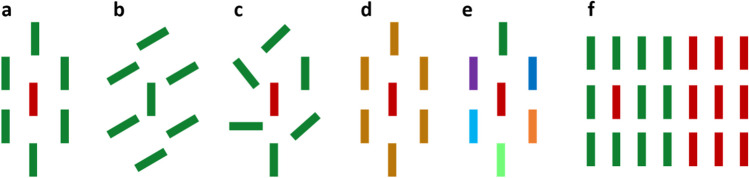
Illustrations of different types of singletons

It can be useful to differentiate between **relative** and **absolute features**. Absolute features are all those features that a stimulus possesses irrespective of what other stimuli appear in the display. For example, a red stimulus possesses the absolute feature RED independent of whether the stimuli surrounding it are red or green. By contrast, **relative** features (e.g., “redder”) depend on the absolute features of other stimuli in the display – the visual context. Stimulus **salience** is relative, determined by local feature contrast (Nothdurft, 1992; see also *texton gradient*, Julesz, 1986) via mechanisms of *iso-feature suppression* and *lateral inhibition*. Relatedly, “singletonness” also refers to a relative feature, with a singleton being defined as the only stimulus with a particular absolute feature among other stimuli. The central bars in Fig. 3a–c are clear examples of singletons. The one in Fig. 3c, for instance, is a singleton in color but not in orientation. Figure 3d–f illustrate less straightforward cases. The central bar in Fig. 3d is a singleton, but is not particularly salient. This is worth noting because, in many papers, singletons are assumed to be salient. In Fig. 3e, the central stimulus is the only red stimulus, but as all other stimuli have their unique color as well, most researchers will not refer to it as a singleton. Finally, in Fig. 3f, the single red stimulus on the left is a singleton locally (in the left part of the display) but not globally (across the whole display). The terms **first-order** versus **second-order features** have been used to refer to absolute versus relative features of singletons (Gaspelin & Luck, 2018a), but these terms are not to be confused with *first- versus second-order image statistics* (Julesz, 1975; Lu & Sperling, 2001).

A stimulus either is or is not a singleton. In contrast, salience is a continuous quantity, governed by complex rules, many of which have not been fully worked out empirically. For example, it would be tempting to think that salience could be defined by some distance in a feature space, but this is not necessarily so. So-called “search asymmetries” are a clear example of this. For instance, search for a line tilted 18° from vertical among vertical distractors is easier than search for a vertical target among 18° distractors, even though, of course, the 18° difference between target and distractors is the same in both cases (Treisman & Gormican, 1988).

Empirically, salience can be measured by having participants use a reference stimulus in one dimension to match the salience of a test stimulus in another (Nothdurft, 2000) or by measuring how long it takes to find a target defined by a feature among homogeneous nontargets (Zehetleitner et al., 2013). For example, if a *red* vertical target is found faster than a green *horizontal* target in search for a unique target among green vertical nontargets, one would conclude that *red* is more salient than *horizontal* among these nontargets. This approach has been used to verify that a given singleton distractor is more salient than the target in an additional-singleton task (→ ***Tasks***; e.g., Liesefeld et al., 2022). Salience can also be estimated using computational salience models (Itti & Koch, 2000), and some researchers have applied such models to determine the salience of distractors (Chang et al., 2021; Stilwell & Gaspelin, 2021). Inconveniently, computational (typically pixel-based) models often do not perform as expected on laboratory stimuli (Kotseruba et al., 2021; see Wischnewski et al., 2010, and Jeck et al., 2019, for more promising, proto-object-based approaches) and, indeed, it is not clear whether the behavioral methods agree with each other. Simply put, developing consensual measures of salience has been surprisingly arduous. This will be an important issue for future research.

### Tasks

#### Terms

Task, detection task, localization task, discrimination task, search performance.

A **task** refers to what participants are instructed to do, for instance, search for a given letter and report its color (Fig. 2). In **detection tasks**, participants are required to respond as to whether a pre-specified target (e.g., a red stimulus) is present or absent. In **localization tasks**, participants are required to find the target and respond to its location with various degrees of precision: for instance, they may be asked to determine whether the target appeared on the right or left of fixation or to click the computer mouse at the exact location of the target. In **discrimination tasks**, also termed *identification*, *classification*, or *compound* tasks, the target has one of two or more possible reported features (→ ***Features***) on each trial (for instance the red target may enclose either a horizontal or a vertical line) and participants are required to find the target and classify its reported feature. In the context of a psychological experiment, *performance* refers to response times and/or task accuracy. In the majority of visual-search studies, including most studies on visual distraction, **search performance** more specifically refers to the time needed to correctly respond to target presence (detection tasks) or to the target’s reported (discrimination tasks).

### Paradigms

#### Terms

Paradigm, additional-singleton paradigm, distractor interference, split-block paradigm, dot-probe paradigm, probe-detection performance, letter-probe paradigm, spatial cueing paradigm, contingent-capture paradigm, cue-validity effect, contingent-capture effect, attentional-blink paradigm, spatial-blink paradigm, irrelevant-singleton paradigm, distractor-cueing paradigm

In the study of distraction, a **paradigm** refers to an experimental procedure that is characterized by a core manipulation and a core comparison of interest, although its variants may differ in many respects. In particular, the same paradigm can be used with different tasks: with the spatial-cueing paradigm, for instance, observers might be asked to detect the target or to discriminate it (→ ***Tasks***). Thus, although the term *task* is often used to refer to a paradigm (e.g., spatial cueing task), we recommend reserving this term to refer to what participants are instructed to do.

**Fig. 4:**
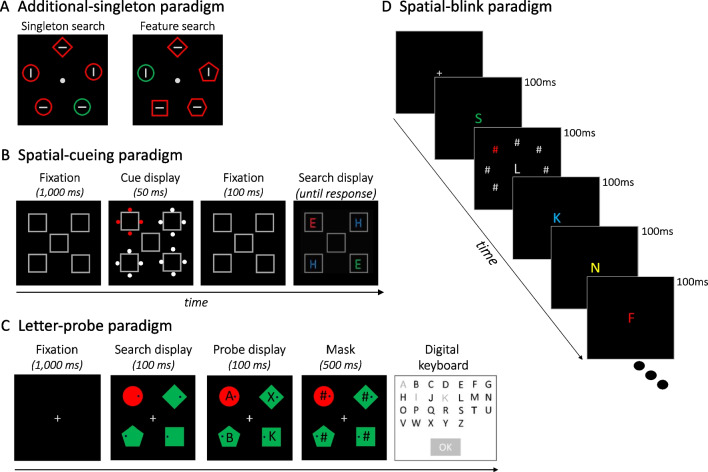
Illustrations of different types of paradigms. **Panel A:** The target is the unique diamond, among either homogeneous shapes (singleton search) or heterogeneous shapes (feature search), and the distractor is the unique green item. **Panel B:** The target is the red letter, and the cue is the red dot set. **Panel C:** The sample trial is a probe trial, the target is the diamond, and the distractor is the red item. **Panel D:** The target is the red letter, and the distractor is the red #-sign. Display durations are for illustration purposes and vary among studies. See text for additional information

In the standard version (Theeuwes, 1992) of the **additional-singleton paradigm**, search displays consist of a variable number of shapes presented on an imaginary circle around fixation (see Fig. 4A, Singleton search), each of which encloses a small line, the response feature. Participants are required to make a discrimination response to the line inside the target (e.g., horizontal or vertical; *discrimination task* → ***Tasks***). The target is defined by its unique shape and is typically a singleton (→ ***Features***) among homogenous nontargets (→ ***Stimuli***, e.g., the diamond amongst circles). The critical manipulation is that on some trials (typically 50%) one of the irrelevant items has a unique color (e.g., it is the only red stimulus among green stimuli), and is referred to as the *color-singleton distractor* (→ ***Features***). The comparison of interest is between search performance on distractor-present trials versus distractor-absent trials, which indicates *distractor-presence costs* or **distractor interference** (→ ***Types of distraction***) when the presence of the distractor impairs search performance, and *distractor-presence benefits*, when the presence of the distractor improves search performance. Distractor-presence costs are typically held to indicate that the distractor captured attention, whereas distractor-presence benefits are typically held to indicate that the distractor was suppressed (but see Lamy, 2021, for a criticism against inferring mechanisms from net effects).

In variants of the additional-singleton paradigm, the proportion of distractor-present trials, the dimensions on which the target and singleton-distractor are defined and whether they are defined on the same or on different dimensions, the response feature, as well as the spatial lay-out (e.g., a grid rather than a circular display) may change.

In a particularly often used variant of the paradigm, the target appears among heterogeneous nontargets (see Fig. 4A, Feature search) and it is therefore not a singleton against a homogenous background of non-targets (Bacon & Egeth, 1994; → ***Stimuli***). This variant is often referred to as the *feature-search variant* (→ ***Templates and strategies***) of the additional-singleton paradigm, even though in this variant, some would consider only the distractor, but not the target, a singleton (→ ***Features***), so that, strictly speaking, there is only one singleton. In another increasingly popular variant of the additional-singleton paradigm, the **split-block paradigm**, the color of the singleton distractor changes on each block of trials (e.g., Vatterott & Vecera, 2012). The comparison of interest is the distractor interference/benefit before versus after participants gain experience with the singleton distractor’s color, that is, early versus late during each block. Finding that distractor interference is reduced or even becomes a benefit after several exposures to the same distractor feature is held to indicate that this feature becomes suppressed.

The typical **dot-probe paradigm** used to study distraction (Kim & Cave, 1999) is similar to the additional-singleton paradigm except that it is a *detection task* (→ ***Tasks*)**, the search display is presented only briefly and, on a proportion of the trials, it is followed by a small dot after a time interval that can vary within or across studies. The dot appears randomly at the location previously occupied by one of the search-display stimuli. At the end of each trial, participants are required to indicate whether the target was present or absent, but when the dot is present, they have to respond to its onset as fast as possible before responding to the presence or absence of the target. The typical comparison of interest is response time to the dot onset (**probe-detection performance**) when it appears at the location of the distractor versus that of other stimuli (the target, a non-target). The object at the location of which the dot is responded fastest is held to enjoy the highest attentional priority at the specific moment when the dot appeared following search display onset.

The **letter-probe paradigm** (see Fig. 4C; also referred to as *capture-probe paradigm*; Gaspelin et al., 2015; see also, Kim & Cave, 1995, Exp. 2) is conceptually similar to the dot-probe paradigm. It consists of search trials (typically two-thirds of the trials) and probe trials (one-third of the trials), randomly intermixed. On search trials, observers perform the an additional-singleton task. On probe trials, a letter-probe display appears briefly and is followed by a mask. In this display, a letter is superimposed on each search stimulus, or sometimes on a subset of these stimuli. Participants are required to report the letters they saw. The critical measure is the percentage of correctly reported letters, which serves as an index of attentional priority. The comparison of interest is the difference in the percentage of correctly reported letters at the critical distractor’s location relative to the average across non-target locations and sometimes also relative to the target location. Finding that more letters are reported from the distractor’s location than from nontarget locations is taken to indicate that the distractor captured attention, whereas the opposite pattern, that is, fewer reported letters, is held to indicate that the distractor was suppressed (but see Lamy, 2021, for a criticism against inferring mechanisms from net effects). With appropriate modifications (e.g., a forced-choice between two possible probe target-letters) it is reasonable to analyze reaction time as well as accuracy (e.g., Chang & Egeth, 2019).

Building on Posner’s (1980) classical exogenous cueing paradigm, the typical **spatial cueing paradigm** used to study distraction in visual search (e.g., Folk et al., 1992; see Fig. 4B) requires participants to search for a pre-defined target in a search display (e.g., the red stimulus among three gray stimuli) and to respond to its identity (e.g., “=” or “x”). Shortly prior to the search display onset a cueing display appears briefly. It includes a *singleton* (→ ***Features***) that appears randomly at the location of one of the search stimuli. The comparison of interest is *search performance* (→ ***Tasks***) on validly cued trials, that is, trials in which the target appears at the location of the cue versus on invalidly cued trials, that is, trials in which the target appears at a different location). The difference in performance between valid- and invalid-cue trials is referred to as the **cue-validity effect**. In the most popular version of this paradigm, the **contingent-capture paradigm**, the match between the cue’s salient feature and the target-defining feature is manipulated. For instance, a red singleton cue in search for a red target is referred to as a *matching* or *relevant-color* cue, whereas a green singleton cue is referred to as a *non-matching* cue or *irrelevant-color* cue. The comparison of interest, referred to as the **contingent-capture effect**, is the difference between the *cue-validity effect* elicited by matching versus non-matching cues. Such a difference, when found, is taken to index the impact of search goals on attentional priority.

In the typical **attentional-blink paradigm**, a rapid stream of stimuli is presented at fixation, and either one or two targets can appear within the stream (e.g., Broadbent & Broadbent, 1987). The task is to report these targets at the end of each stream/trial. The attentional-blink (AB) effect refers to poorer identification performance when the second target (T2) appears soon after the presentation of a correctly identified first target (T1), typically between 200 and 700 ms (an interval referred to as the *AB period*) than when it appears later. That is, poorer T2 performance indicates that T2 was presented in the blink period induced by T1. This impairment in T2 processing is generally assumed to be a consequence of attentional selection of T1 (but see Ophir et al., 2020).

In the variants of the attentional-blink paradigm used to study distraction T1 is replaced with a distractor, typically a color singleton. In this case, the distractor is therefore a singleton in time rather than in space. In one variant referred to as the *spatial-blink paradigm* (Folk et al., 2002; see Fig. 4D), the RSVP stream in which the target appears is presented at fixation, and a singleton distractor is presented at a peripheral location. In another variant, the singleton distractor appears prior to the target within the same stream, that is, also at fixation. The core manipulation is the time between the distractor and target onsets. If the distractor creates a blink, that is, if identification performance is poorer when the target appears within the blink period than outside of it, it is inferred that the distractor captured attention. Note, however, that when the distractor appears at fixation, capture does not manifest in an attentional shift, *attentional capture* → ***Types of distraction***, but in what is often called *attentional engagement* (Folk et al., 2009; Zivony & Lamy, 2018).

In a typical **irrelevant-singleton paradigm** (e.g., Yantis & Egeth, 1999), participants search for a target defined by its shape (e.g., a specific letter) among a variable number *n* of nontargets (*set size* → ***Stimuli***) and have to report whether it is present or absent (*detection task* → ***Tasks***). All stimuli have the same color except for one, the color-singleton distractor. The positions of the target and of the color singleton are independently and randomly selected, so that they have a 1/*n* chance to overlap on target-present trials. In other words, the target is the color singleton on 1/*n* of the trials. The comparison of interest is the difference in search slopes when the target is the color singleton relative to when it is not. Finding that search slopes are flatter in the former condition is taken to indicate that the color singleton enjoys more priority than the nontargets (but for criticisms of this interpretation of search slopes, see Christie et al., 2014; Kristjánsson, 2015).

In a typical **distractor-cueing paradigm** (e.g., Arita et al., 2012), the target, defined by its shape (e.g., an upright or an inverted T), appears among non-targets and its color can vary from trial to trial. On each trial, a pre-cue appears and the core manipulation is whether this cue is negative or neutral, but positive cues are also usually included. A negative cue indicates a feature that does not characterize the target; therefore, if this cue can be used to deprioritize stimuli with this feature, it should help participants reduce the number of possible candidate stimuli competing for attention. A neutral cue provides no information, whereas a positive cue indicates the probable feature of the target. The comparison of interest is search performance with negative versus neutral cues. Finding that the target is responded to faster on negative-cue versus neutral-cue trials is held to indicate that features known to characterize distractors can be purposefully and proactively suppressed (→ ***Timing***).

#### Types of distraction

##### Terms

Distraction, attentional capture, filtering costs, distractor interference, response-compatibility effects

According to the Collins dictionary, “distraction is something that turns your attention away from something you want to concentrate on.” In the attentional literature, distraction refers to the *fact* that your attention is taken away rather than to the *thing* that takes it away - which is referred to as a *distractor* (→ ***Stimuli***). Distraction may manifest in two qualitatively different ways. First, distraction may manifest as attentional shifts towards a location or feature that is task-irrelevant (a *distractor* → ***Stimuli***). In this case, attention is said to be *captured* by the distractor (**attentional capture)**. Second, distraction may manifest as a delay in directing attention to the relevant stimulus (*target* → ***Stimuli***) when the distractor is present, although attention was never actually allocated to the distractor. Distraction in the absence of attentional capture is sometimes referred to as **filtering costs** (Kahneman et al., 1983; see also Folk & Remington, 1998). In the context of the *additional-singleton paradigm* (→ ***Paradigms***), attentional capture was originally suggested as the mechanism underlying the increased response times on distractor-present relative to distractor-absent trials (Theeuwes, 1992). However, Folk et al. (1992) objected that impaired *search performance* (→ ***Tasks*)** on distractor-present trials (*distractor-presence costs* or **distractor interference**) do not prove that attention was actually shifted to or captured by the distractor. Instead, the longer response times may result from filtering costs.

To provide convincing evidence for attentional capture, it is therefore necessary to track the spatial locus of attention and show that attention was allocated to the distractor. Currently, there are two broad types of measures allowing for the localization of attention. First, measures showing that attention was focused on the distractor location, such as *response-compatibility* effects, the proportion of overt shifts of attention (i.e., eye movements) made to the distractor relative to non-targets and distractor-elicited ERP components associated with attentional selection (e.g., N2pc; Eimer & Kiss, 2007; Hickey et al., 2006; see also ***Timing***). **Response-compatibility effects** are measured in discrimination tasks where identifying the reported feature requires focused attention. In the distraction literature (e.g., Theeuwes & Burger, 1998; Carmel & Lamy, 2014), they typically refer to better performance when the reported feature of the target requires the same motor response as the reported feature of the distractor (compatible trials) relative to when it requires a different motor response (incompatible trials). Second, measures showing that identification of stimuli presented briefly after the distractor is better when these stimuli appear at the distractor location. For instance, cue-validity effects in the *spatial cueing paradigm*, probe-detection performance in the *dot-probe paradigm*, and the percentage of correctly reported letters in the *letter-probe paradigm* all belong to this category (→ ***Paradigms***).

#### Templates and strategies

##### Terms

Target template, negative template, attentional control setting, attentional strategy, singleton-detection mode, feature-search mode, optimal tuning of attention, relational coding, dimension weighting

Duncan and Humphreys (1989) introduced the concept of an *internal template*, or **target template**, to designate features of the target that are represented in memory. The presumed function of a target template is twofold. On the one hand, it provides features to guide attention by increasing sensory gain or attentional priority (*search-guiding features* → ***Features***) and on the other hand, it serves to determine whether a selected stimulus is indeed the target (*target-defining features* → ***Features***). Typically, templates are conceived as positive in the sense that they contain information about the target(s). However, templates might also be negative, when they contain information about the distractors (e.g., Woodman & Luck, 2007). As such, **negative templates** might be used to reject a subset of the distractors and thereby facilitate search (e.g., Arita et al., 2012). However, the idea that negative templates can guide attention is debated. Some authors suggest that information about distractor features is recoded into positive templates (e.g., Beck & Hollingworth, 2015) or that, contrary to target enhancement, distractor suppression is not under voluntary control, but relies only on repeated experience with the distracting information (for reviews, see Noonan et al., 2018; van Moorselaar & Slagter, 2020).

Although search-guiding templates are often thought to exactly correspond to the actual target features, they may deviate in various ways (e.g., Geng & Witkowski, 2019). First, when the target can be redundantly distinguished from the non-targets by two features, participants may choose to include just one in their template. For instance, when the target is reliably the unique circle among diamonds, participants may adopt the **attentional strategy** (Leber & Irons, 2019) to search for unique or salient stimuli (**singleton-detection mode**, Bacon & Egeth, 1994) instead of using “circle” as their search-guiding template (**feature-search mode**). Second, when the similarity between target and distractors is high, templates may be adapted away from the actual target feature to increase the efficiency of search through **optimal tuning of attention** (Navalpakkam & Itti, 2007). That is, the search-guiding features stored in the template are shifted away from distractor features to avoid overlap. Third, when the relation between the target feature and the distractors’ features is stable, this may be reflected within the search template. Finally, the search-guiding template may be coarser than the target-defining template (Kerzel, 2019).

Note that some authors do not adhere to the idea that visual search is guided by stored visual representations (Becker et al., 2019; Liesefeld et al., 2019, 2022). For instance, Becker et al. (2013) suggested a **relational coding** mechanism: when the target is the red stimulus among orange distractors, the search-guiding template would include the relative feature “redder” rather than “red” and, as a result, an orange stimulus among yellow ones would attract attention. **Dimension weighting** (e.g., Liesefeld & Müller, 2019) refers to the up- or down-modulation of salience signals (→ ***Features***). In particular, the Dimension Weighting Account postulates that the weighting of salience signals during integration on the overall priority map (→ ***Priority map***) is affected by goals and selection history (→ ***Guidance***).

It is commonly assumed that templates are stored in visual working memory; they may be coded actively through persistent neural firing (Chelazzi et al., 1993; Funahashi et al., 1989) or remain latent through changes in synaptic weights (Stokes, 2015; Wolff et al., 2015). However, templates may be stored in long-term memory, in certain conditions (e.g., Carlisle et al., 2011; Wolfe, 2021). The contents of templates have been probed both directly and indirectly. To probe the search-guiding template directly, established techniques from the memory literature have been used. For example, when the main task is to search for a color target, participants may be unpredictably asked on a subset of the trials to indicate its color (Navalpakkam & Itti, 2007). To probe the search-guiding template indirectly, the *contingent-capture paradigm* has been used extensively (→ ***Paradigms***). The core assumption is that attention is more robustly captured by a distracting feature the more closely this feature matches the observer’s search-guiding template. In that context, the search-guiding template is often referred to as the **attentional control setting** or *attentional set*, although that term also encompasses other task-relevant information not stored in the target template (e.g., temporal information, relative features → ***Features***).

#### Priority map

##### Terms

Priority map, salience map, conspicuity map, dimensional map, overall (salience or priority) map

The primate visual system is organized topographically with multiple retinotopic maps in cortical and subcortical structures. Because visual spatial attention is manifest in and through such a visual system, it is natural to employ the concept of a map or a set of hierarchical maps in explanations of how attention is allocated. We suggest that **priority map** should be used as the most generic, theoretically neutral description of a single map or, alternatively, a map at the top of the hierarchy that serves to prioritize some visual information over other. It should replace terms such as *activation map* that has been used in the context of *Guided Search* (Wolfe, 1994) or *master map of locations* that has been used in the context of *Feature-Integration Theory* (Treisman & Souther, 1985). **Salience map** should be reserved for maps that index exclusively the feedforward, bottom-up, retinal-based, stimulus-driven representation of visual distinctiveness of a feature, stimulus, or location (i.e., *salience* → ***Features***). Thus, if a theory assumes that information is prioritized only due to bottom-up influences, they postulate a salience map, whereas theories acknowledging additional influences such as task relevance and prior history (→ ***Guidance***) postulate a priority map. Computational models derive salience from the local contrast in the spatial distribution of various features across the display (→ ***Features***). It appears likely that salience maps, if they exist at all in a pure form, reside in relatively early visual brain regions, or other sensory regions for other modalities, and that priority maps reside at higher processing levels.

Attentional priority refers to the relative strength of activation at each region of the priority map. Attentional priority is a dynamic representation of the combination of a variety of influences that have different time courses, so that the activation landscape changes across time (→ ***Timing***). More specifically, the activation at each point in time and space, that is, the output of priority computations, signals a quantity proportional to some weighted combination of stipulated influences (→ ***Guidance***). Points in the maps might represent raw location or they might index something akin to objects (*proto-objects*; Jeck et al., 2019; Wischnewski et al., 2010). Because salience and priority maps are derived from visual processing embodied in the representation of the visual field, it is reasonable to assume that they are homeomorphic with the map of the visual field. Accurate models of these maps should therefore take magnification of the central visual field and other distortions into account. Variation in resolution of attention deployment across the visual field might be a by-product of visual processing or a distinct feature of the extraretinal processes guiding attention (Hulleman & Olivers, 2017).

In most models of visual attention and search, the amount of activation determines where attention or gaze is allocated next (Wolfe, 2021) or the likelihood that such a shift of overt or covert attention will be executed (Moran et al., 2013), an assumption that implies a winner-takes-all mechanism and sequential attention allocations. Other models assume that priority map activation determines the degree to which stimuli at the respective locations are processed in parallel, that is, directly and not via an attention allocation (Bundesen, 1990). Models also differ in whether priority is thought to guide categorization of a feature or a stimulus (“decide that”) or to guide selection-for-action through response preparation (“decide to”). If not further specified or contextualized, “priority map” refers to the single map guiding attention allocations, and possibly gaze shifts mediated via attention, but if there are multiple priority maps as suggested by some investigators based on neural findings (Bisley & Mirpour, 2019), these might serve different purposes. For instance, a priority map in one cortical area might specify covert attention allocation, while a priority map in another cortical area might specify overt gaze shifts or hand movements, directly rather than via attentional mediation.

**Fig. 5:**
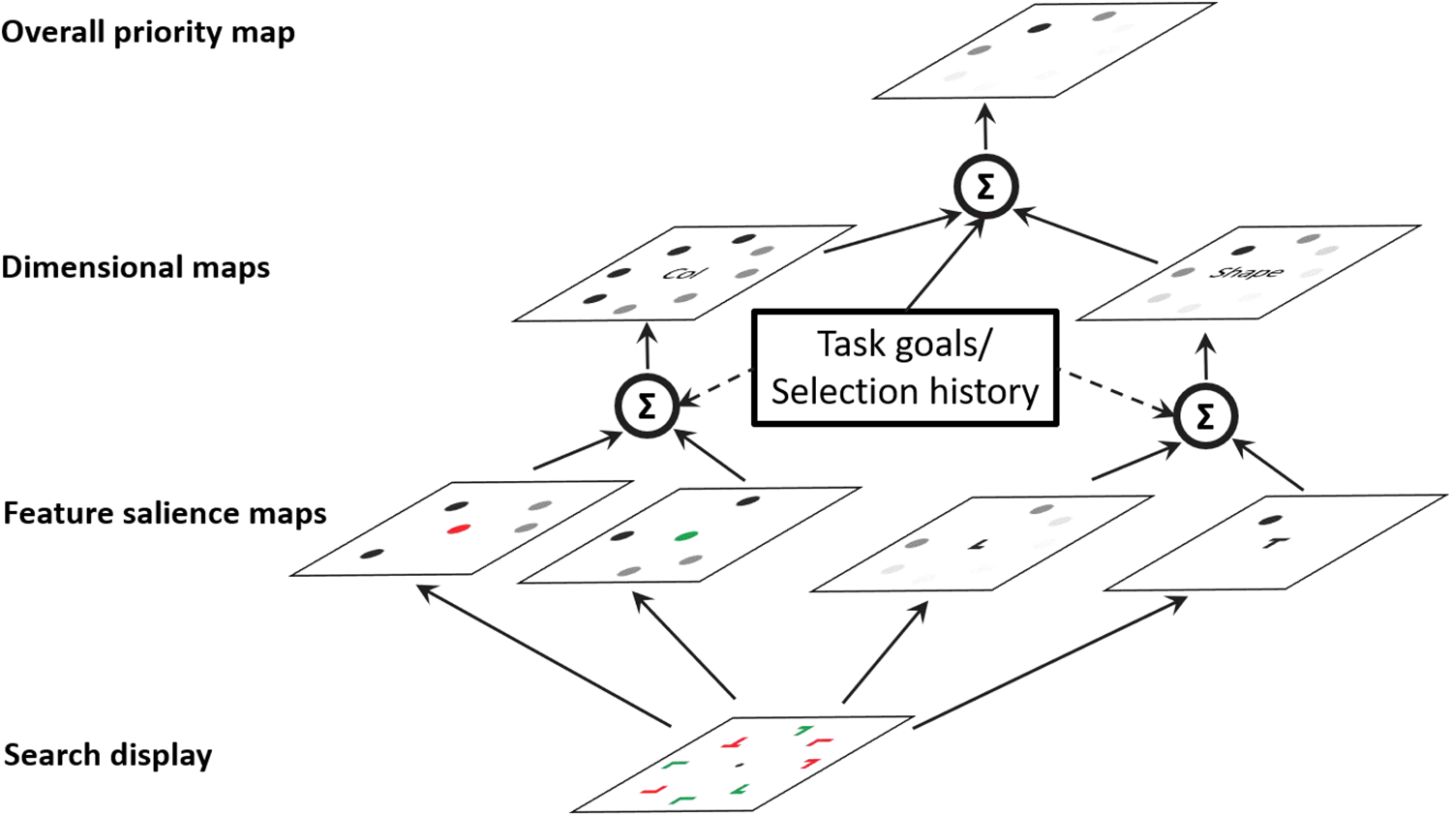
Illustration of a schematic architecture illustrating the concepts introduced in the text. At the input level, features are extracted and iso-feature suppression contributes to salience computation (→ *Features*). The output can be combined at an intermediate dimensional level. Ultimately, the most conspicuous stimuli across features and dimensions are represented. In the overall priority map the representation of conspicuous stimuli is combined with or influenced by task goals and selection history (→ ***Guidance***). Some investigators think that this influence can happen at lower levels of this hierarchy, indicated here by dashed lines

Some models stipulate a hierarchy of maps, where dimension-specific spatial representations referred to as **conspicuity maps** (Itti & Koch, 2000) or **dimensional maps** (Liesefeld et al., 2018) form an intermediate level between feature maps and a cross-dimensional or **overall (salience or priority) map** (see Fig. 5).

#### Guidance

##### Terms

Guidance, stimulus-driven, bottom-up, top-down, goal-directed, selection history, feature/space/time-based control of attention, statistical learning, value learning, intertrial priming, scene guidance

Our information processing capacity is limited and our motor behavior can only be directed to a limited subset of stimuli. Such limitations require that we prioritize some stimuli in our environment over others. This prioritization process is usually conceptualized as the resolution of the competition among the locations or stimuli in our visual field, represented on an overall priority map (→ ***Priority map***). Attention is then assumed to be allocated to the location(s) with the highest activation on the priority map, in a winner-take-all fashion (but see Jiang, 2018, who suggests that search habits may affect attention allocations via a different route).

The study of attentional **guidance** focuses on the factors that modulate activations on the overall priority map. Several sources of attentional guidance have been distinguished, among which are visual salience, observers’ goals, selection history, and scene guidance. These sources are thought to jointly influence activations on the priority map. Therefore, the peak on this map depends on the relative strengths of the different guiding factors at any given moment. Note that while this peak is often thought to determine where attention will be shifted next, other suggestions have been put forward (see, e.g., Moran et al., 2013, for a probabilistic relation between priority and attention allocations; Yaron & Lamy, 2021, for the idea that contextual information constrains when the next shift of attention to the peak on the priority map is triggered).

Stimuli that are visually salient (→ ***Features***) are endowed with higher priority weights than low-salience stimuli, resulting in **stimulus-driven** guidance. When attention is directed to a high-salience yet irrelevant stimulus and salience therefore overrides other sources of attentional guidance, one speaks of stimulus-driven (or **bottom-up**) *capture of attention* (→ ***Types of distraction***).

In contrast to stimulus-driven guidance, **top-down** guidance effects are driven by the internal states of the observer and have a strong impact on the priority map. While it is uncontroversial that goal-directed guidance is an instance of top-down control, many researchers identify selection history effects as a collection of factors that guide attention, but do not fit squarely within the classic top-down and bottom-up dichotomy. Because they view top-down guidance as intentional, whereas selection-history effects are thought to be implicit, these researchers feel that selection history should be treated as a separate source of guidance (e.g., Awh et al., 2012). By contrast, other researchers rely on the definition of top-down processes in perception research, namely “knowledge, expectations, or past experiences that shape the interpretation of sensory information” (Gazzaniga et al., 2016). Accordingly, they think that selection history should be grouped with goal-directed guidance under top-down control because both are driven by internal states of the observer (e.g., Egeth, 2018; Gaspelin & Luck, 2018c; Liesefeld & Müller, 2021; Ramgir & Lamy, 2022). This controversy is mainly semantic, though, because there is a relatively wide consensus around the idea that goal-directed guidance should be distinguished from selection history.

**Goal-directed** guidance**.** Observers can direct their attention to a location in space where they expect a goal-relevant stimulus to appear (**space-based control of attention**) or to a subset of stimuli possessing a known goal-relevant feature (**feature-based control of attention**). It is often assumed that this is achieved by using information stored in memory to set up a template of the relevant feature in working memory (*search-guiding template* → ***Templates and strategies***). Accordingly, distractors matching the search-guiding template may capture attention (→ ***Types of distraction***; *contingent capture* → ***Paradigms***). This also occurs when a feature is held in working memory for a memory test: attention is biased towards stimuli matching that feature in an unrelated search task that intervenes during the retention interval (e.g., Downing, 2000). Attention can also be directed to a certain moment in time (*time-based control of attention*; e.g., Coull & Nobre, 1998). In addition, when the target can be defined as a *singleton* (→ ***Features***), observers can adopt a singleton-detection mode (→ ***Templates and strategies***) and purposefully search for any visually salient stimulus (Bacon & Egeth, 1994; Leber & Egeth, 2006) or for a salient stimulus in a particular feature dimension (dimension-weighting account; Found & Müller, 1996; Liesefeld et al., 2022; Liesefeld & Müller, 2019; Müller et al., 1995). Goal-directed guidance is thought to be explicit (observers are aware of the goal-relevant property), flexible (observers can cease to prioritize a location or feature if it no longer serves their goals), and proactive (→ ***Timing***). While it is established that goal-directed guidance can increase the weights of the locations matching the relevant property on the priority map (*attentional enhancement* → ***Modulation***), the notion of *negative templates* (→ ***Templates and strategies***) has gathered less empirical support (for evidence in favor, see Arita et al., 2012; Munneke et al., 2008; for evidence against, see Reeder et al., 2018; van Moorselaar & Slagter, 2019; for a review, see van Moorselaar & Slagter, 2020).

**Selection history** refers to a heterogeneous collection of mechanisms (Anderson et al., 2021), which have in common the fact that they cannot be categorized as stimulus-driven or goal-directed, and that they depend on past experience. Observers are often not aware that these mechanisms are influencing their attention, and selection-history mechanisms are therefore typically considered to be implicit. The most widely investigated selection-history phenomena can be grouped into three main categories: statistical learning, value learning and inter-trial priming.

**Statistical learning** refers to the finding that statistical regularities with regard to the locations, features or temporal characteristics of targets and/or distractors influence attentional priority (e.g., Geng & Behrmann, 2002, 2005). For instance, if a salient distractor appears at a given location substantially more often than would be expected by chance, observers will ignore this distractor more efficiently when it appears at the high-probability location than elsewhere (Ferrante et al., 2018; Goschy et al., 2014; Sauter et al., 2018; Wang & Theeuwes, 2018a, 2018b). Conversely, under certain conditions, they will have more difficulty selecting the target when it appears at the high-probability distractor location than elsewhere (Allenmark et al., 2019; Sauter et al., 2018; B. Zhang et al., 2019a).

**Value learning** refers to the finding that stimuli that are reliably associated with either reward or aversive outcomes are endowed with higher attentional priority than neutral-value stimuli (Anderson et al., 2011). For instance, if red targets are repeatedly associated with a higher reward than green targets, a red distractor will cause more distraction (→ ***Types of distraction***) than a green distractor in subsequent search for a shape-defined target.

Finally, **inter-trial priming** refers to the finding that when a stimulus was selected as a target on a previous trial, attention is biased towards subsequent stimuli sharing a property of that target – for instance, one of its basic features, such as its color or shape (*feature intertrial priming*, aka *priming of pop-out* or *PoP*, Maljkovic & Nakayama, 1994), some more complex property such as a face’s emotional expression (Lamy, Amunts, & Bar-Haim, 2008a), its location (*location intertrial priming*, aka *priming of location* or *PoL*, Maljkovic & Nakayama, 1996), or its being a *singleton* (→ ***Features***) on the same dimension (*dimension inter-trial priming*, Found & Müller, 1996; see also Lamy, Bar-Anan, & Egeth 2008c; Won et al., 2019). Similar processes can bias attention away from stimuli sharing a property with recently rejected distractors (e.g., Kristjánsson & Driver, 2008; Lamy, Antebi, et al., 2008b).

**Scene guidance** is a concept closely related to selection history, where a scene can help direct attention during search for a specific target (e.g., Biederman, 1972; Võ et al., 2019; Wolfe, 2021). For instance, when searching for a toaster in a scene depicting a kitchen, search is more likely to start in locations close to the kitchen counter than close to the floor or ceiling. One recent study found that global scene information can also be used to quickly reject nontarget objects and locations, thereby enhancing search efficiency in naturalistic environments (Hickey et al., 2019). Like selection history, the impact of scenes on visual search is likely driven by past experience with scenes and the extraction of statistical regularities in the environment.

It is assumed that a distractor has the potential to capture attention when the combined sources of attentional guidance endow it with high activation on the priority map (→ ***Priority map***). Thus, attentional capture is an outcome of the competition between stimuli in the visual field during a particular task. It implies that no stimulus has the intrinsic power to capture attention (Darnell & Lamy, 2021). To illustrate, a salient stimulus, such as an abrupt onset, may elicit shifts of attention to its location (*attentional capture*; → ***Types of distraction***) more often than a less salient stimulus, such as a no-onset color singleton, but may lose the competition to a color singleton that benefits from other sources of guidance, such as a stimulus that matches the target-defining feature (→ ***Features***) or occurs at the location of a previous target.

To measure the effects of a given source of attentional guidance, some measure of attentional priority is required. There is a large array of indirect measures of attentional guidance, which vary considerably in specificity (e.g., Ramgir & Lamy, 2022), among them, (a) overall search performance (→ ***Tasks***), which is likely to index post-selective processes on top of attentional priority, (b) first eye fixations, often referred to as overt attention allocations, which are thought to provide a direct measure of the relative priority weights that have accrued to each location in the priority map at the moment of saccade execution, and (c) electrophysiological measures thought to index allocation of covert attention (e.g., the N2pc component; Luck & Hillyard, 1994; Luck, 2012, Eimer, 2014; but see Zivony et al., 2018; see also ***Timing***).

While much of the research on attentional guidance has focused on the factors that guide the first shift of attention during a search episode, it is important to remember that guidance operates throughout searches that involve multiple deployments of attention. Different forms of guidance persist differently over time. For example, goal-directed feature guidance can direct attention to many successive stimuli matching the *search-guiding template* (→ ***Templates and strategies***)*,* while bottom-up salience may fade dramatically after the first moments of a search (Donk & van Zoest, 2008; but see Constant & Liesefeld, 2022).

#### Modulation

##### Terms

Inhibition, suppression, attentional suppression, attentional enhancement, enhancement effect, suppression effect.

It has long been proposed that visual attention modulates sensory responses to stimuli based upon their features and locations (→ ***Guidance***). For example, if we are searching a grocery store aisle for a red can of soup, we can restrict attention to red objects to improve the efficiency of our search. Such attentional guidance was initially proposed to occur via enhancement of task-relevant features and locations (Folk et al., 1992; Wolfe, 1994). However, there is now growing evidence that inhibitory processes may also be used to guide attention (Gaspelin & Luck, 2018b; Geng et al., 2019; e.g., via *negative templates* → ***Templates and strategies***).

The idea of **inhibition** is not new to psychology and is often used synonymously with **suppression**. To remain consistent with the ways in which these terms have been used historically, we broadly define them as a voluntary or involuntary restraint that prevents the direct expression of an impulse, action, or thought. Relevant to the current paper, inhibition has also been used to describe reduced visual and attentional processing (Hopf et al., 2006; Reynolds & Heeger, 2009; Störmer & Alvarez, 2014). For example, inhibition has been used to explain reduced perceptual processing of visual information nearby the currently attended region (e.g., *lateral inhibition* → ***Features;*** e.g., Mounts, 2000).

We define **attentional suppression** as any mechanism by which the attentional priority (→ ***Priority map*)** of a stimulus is reduced relative to a situation in which this mechanism is not applied. This suppression could occur based upon the feature, location, or relative timing of the stimulus in question. In contrast, **attentional enhancement** would refer to any mechanism by which the attentional priority of a stimulus is enhanced. It should be highlighted that these two processes are not mutually exclusive: a model could include both suppression and enhancement processes to describe how visual attention is allocated (e.g., Chang & Egeth, 2019; Luck et al., 2021). Some suppressive processes are considered to be more automatic (e.g., *learned distractor rejection*; Gaspelin & Luck, 2018a; Vatterott & Vecera, 2012; Wang & Theeuwes, 2018a, 2018b), whereas others are considered to be more voluntary (e.g., *negative templates* → ***Templates and strategies***; Carlisle, 2022).

**Fig. 6:**
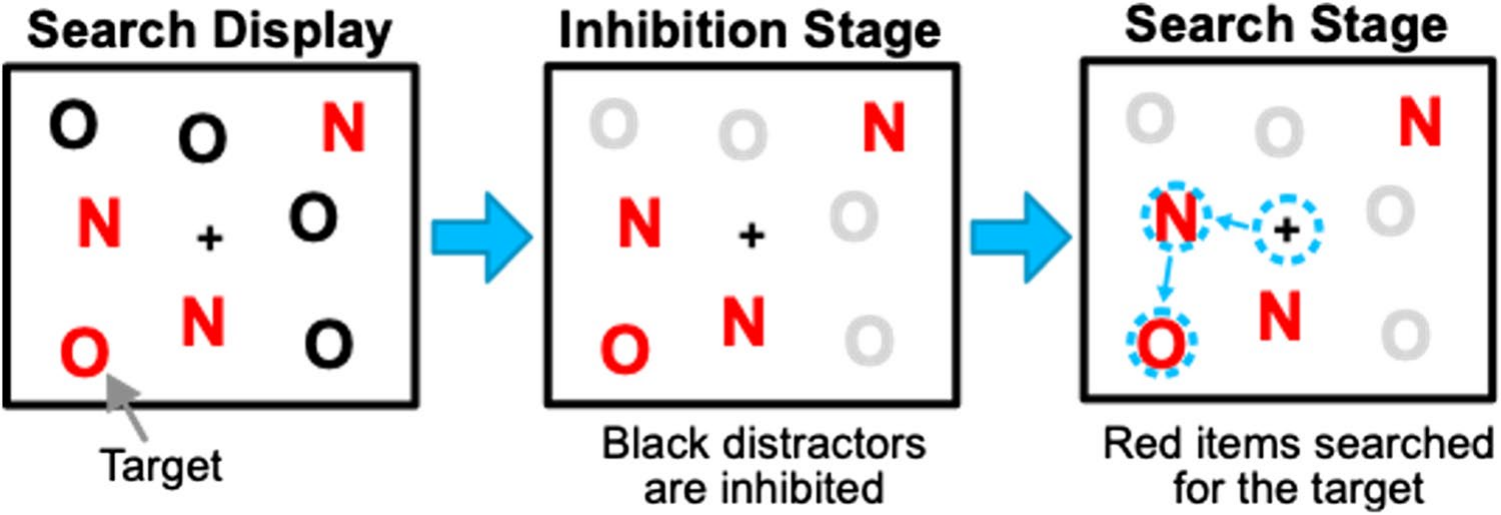
Treisman (1988) used an inhibitory stage to explain conjunction search (stimuli from Egeth et al., 1984). This was later adapted into the feature inhibition hypothesis (Treisman & Sato, 1990)

Many classic models of visual attention have included an inhibitory component. For example, Treisman (1988) used feature-based inhibition to explain observations of shallow search slopes in certain kinds of conjunction searches (e.g., Egeth et al., 1984; Nakayama & Silverman, 1986) – a pattern of results that would seem to refute her feature integration theory of attention (Treisman & Gelade, 1980). To illustrate, consider the conjunction search in Fig. 6, in which participants searched for a red O amongst black O’s and red N’s (Egeth et al., 1984). Treisman (1988) proposed that such a visual search will proceed in two stages. First, participants will segregate the displays into two separate feature dimensions, namely, letter shape and color, and an inhibitory process can be applied to one of those feature dimensions. For example, participants might start by inhibiting distractors with the task-irrelevant color (black) and thereby eliminating these distractors from the search process. Second, the remaining red stimuli will be searched for the target shape. Because it is now a shape singleton amongst the remaining red stimuli and therefore *pops out*, the target will be found quickly, which is in accord with the obtained results. This account was later adapted into the *feature inhibition hypothesis* proposed by Treisman and Sato (1990), which was revised to suggest that multiple feature dimensions could be suppressed in parallel. This is just one of many examples of an attentional theory involving suppression (e.g., Klein, 2000; Logan et al., 2015).

**Fig. 7:**
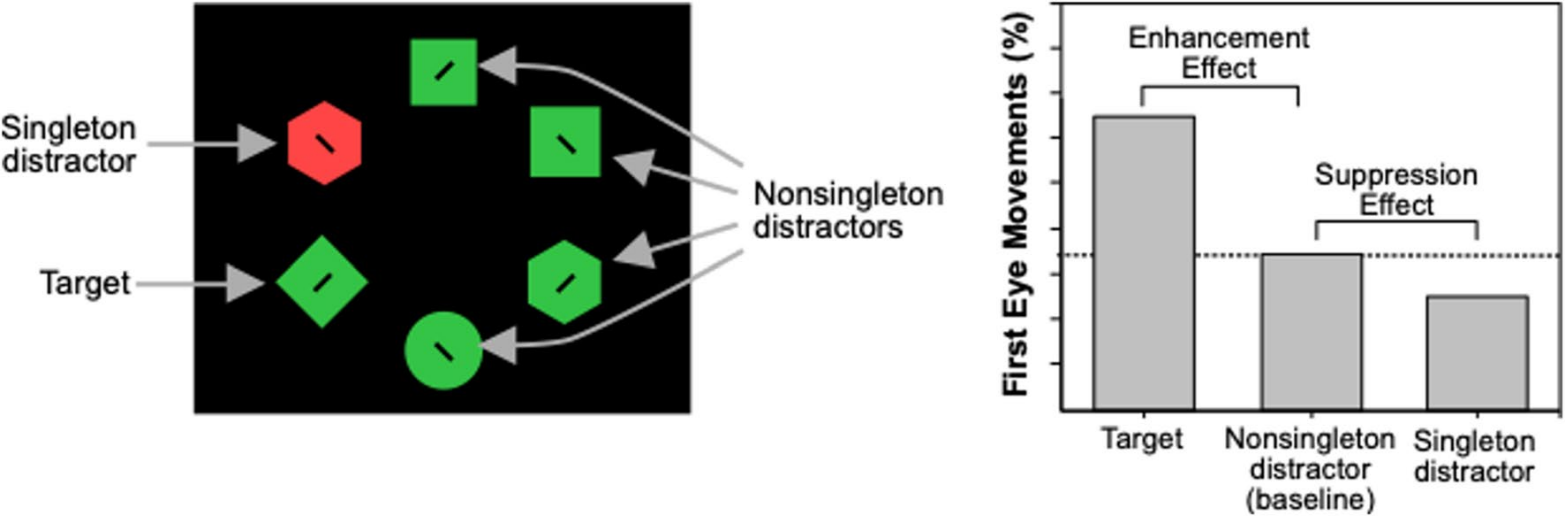
A feature-search variant of the additional-singleton paradigm (→ ***Paradigms***) and hypothetical results demonstrating suppression and enhancement effects on first eye movements (adapted from Gaspelin et al., 2017)

More recently, it has been suggested that attentional suppression plays a crucial role in the ability to ignore salient distractors (→ ***Features;*** → ***Stimuli***). Support for this claim has come from several studies showing that a salient distractor is suppressed below a baseline level (but see Oxner et al., 2022, who criticize the typically employed baseline). For example, Gaspelin et al. (2017) had participants search for a target shape (e.g., green diamond) and ignore a fixed-color *singleton* distractor (Fig. 7; → ***Features***). The destinations of first eye movements were used to evaluate the relative likelihoods that a target, nonsingleton distractor, and singleton distractor were attended. Crucially, the percentage of eye movements to the average nonsingleton distractor was taken as a baseline for comparison with targets and singleton distractors. Targets were *more likely* to attract first eye movements than the baseline level of the average nonsingleton distractor (an **enhancement effect**). Singleton distractors, however, were *less likely* to attract first eye movements than the baseline level (a **suppression effect**). Similar suppression effects have been observed in other studies of eye movements (Adams et al., 2022; Hamblin-Frohman et al., 2022), in probe tasks that measure covert attentional allocation (Chang & Egeth, 2019; Gaspelin et al., 2015; Stilwell & Gaspelin, 2021; Z. Zhang et al., 2019b; → ***Paradigms***), and in single-unit recordings in monkeys (Cosman et al., 2018).

Another approach to demonstrating suppression is to measure a baseline level of a *distraction effect* (→ ***Types of distraction***) and then show that this distraction effect is reduced at a later time point or in a separate experimental condition (Gaspelin & Luck, 2018a, Exp. 4; Müller et al., 2009; Stilwell et al., 2019; Vatterott & Vecera, 2012; Wang & Theeuwes, 2018b; Won et al., 2019; Won & Geng, 2018, 2020). For example, Vatterott and Vecera (2012) had participants perform a task similar to that depicted in Fig. 7, but the color singleton changed on each block. At the beginning of each block, when the singletons had a new color, the singletons produced a singleton-presence cost, indicating that they captured attention. By the end of each block, after participants had learned the singleton’s color, singleton-presence costs were eliminated, suggesting that the singleton’s salient feature was suppressed. Similar learned suppression effects have been found in statistical-learning studies, where salient distractors are more likely to appear in a given location and/or feature than in others (e.g., Chelazzi et al., 2019).

##### Timing

###### Terms

Proactive, reactive, temporal expectations, temporal structure

**Fig. 8:**
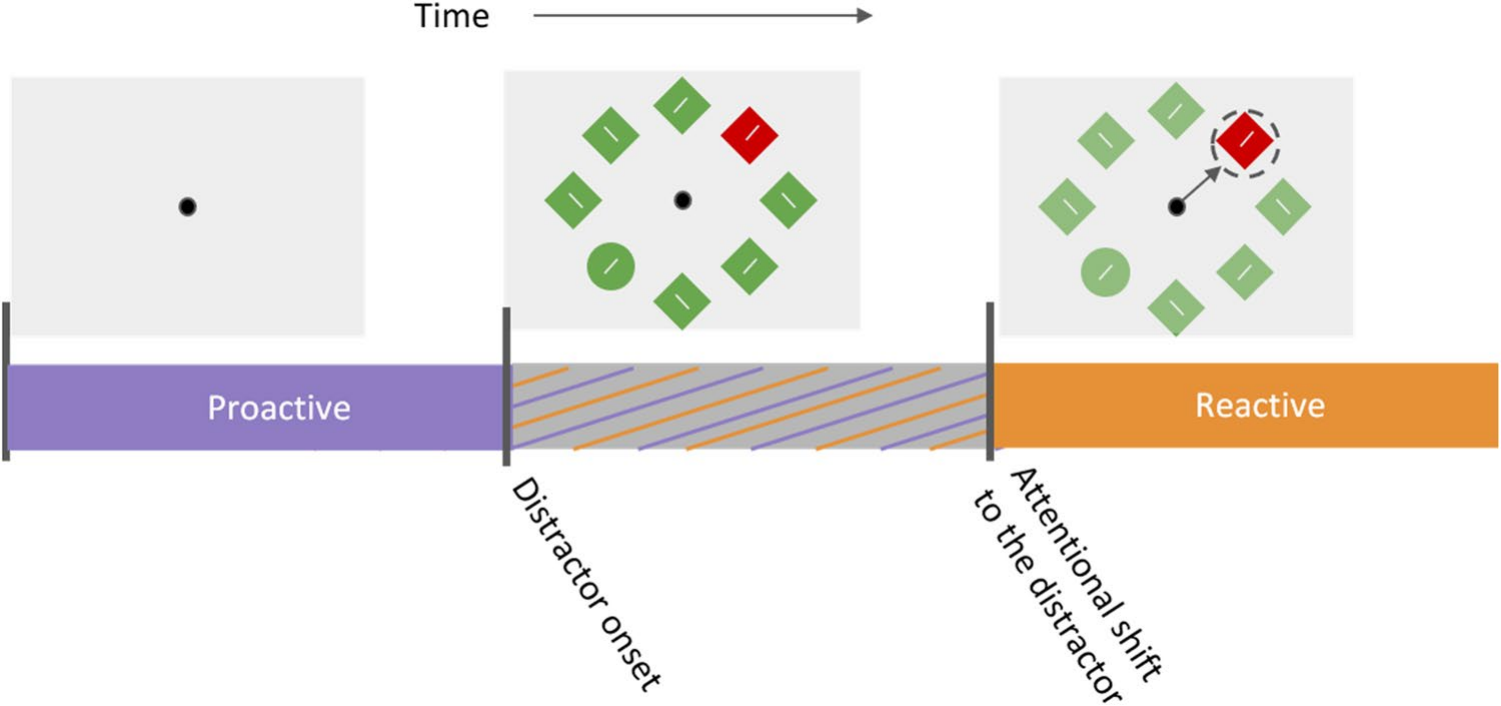
Illustration of the temporal division between proactive (purple) and reactive (orange) suppression. Suppression that is temporally aligned to the immediate onset of the critical distractor is labeled as proactive by some and as reactive by others (see text)

Mechanisms of attention can operate at many points in time to suppress information processing selectively. In laboratory research, the timing of suppressive attentional processes is often described by the terms *proactive* and *reactive* (Braver, 2012; Geng, 2014). However, it has become clear that there is no one shared notion of what the terms *proactive* and *reactive suppression* refer to (Fig. 8). This disagreement is mainly due to the fact that the event of reference is inconsistent across studies. Specifically, *proactive suppression* is often used to refer to suppression that occurs *before* the first shift of attention to reduce (or even prevent) capture by a salient distractor (Estocinova et al., 2016; Gaspelin & Luck, 2018b), but is also sometimes used to refer to a suppressive mechanism that operates *before* the critical distractor appears (e.g., van Moorselaar et al., 2020). Accordingly, *reactive suppression* is sometimes used to refer to suppression that occurs in the aftermath of attentional capture (Luck et al., 2021), but it could also be used to refer to suppression that is triggered by the distractor’s appearance and prevents this distractor from capturing attention (Beck & Hollingworth, 2015). Thus, researchers refer to three different points in time at which suppression might, theoretically, begin (see Fig. 8): (a) before the distractor appears, (b) after the distractor appears but before it captures attention, and (c) after it captures attention. The confusion arises because the intermediate category is labeled “proactive” by some and “reactive” by others. Moreover, this confusion is compounded by the fact that it is often difficult to empirically determine whether suppression occurred at point (a) or (b) and authors who ask whether a distractor can be suppressed before it captures attention are often agnostic as to when exactly the suppression occurred (e.g., Gaspelin & Luck, 2018b; Sawaki & Luck, 2010). To avoid confusion, we suggest that it is important to specify the reference event to which the terms *proactive* and *reactive* are used. In addition, it may be useful to use the term *stimulus-triggered suppression* whenever one specifically refers to suppression that occurs after distractor onset and before attention has shifted to this distractor’s location.

In light of the foregoing discussion, it is uncontroversial that mechanisms are **proactive** if they change baseline brain activity in advance of a stimulus onset. In studies of distractor suppression, there is debate over the reliability of baseline changes in response to expected *distractor* locations and other feature dimensions (van Moorselaar et al., 2020; van Zoest et al., 2021; Wang et al., 2019; see also ***Templates and strategies***). These inconsistencies have led some to question if the mechanisms underlying proactive distractor suppression might differ from those underlying proactive target enhancement, which are more established (Chang & Egeth, 2019; Noonan et al., 2016; van Moorselaar & Slagter, 2019). Proactive suppression may also refer to suppression that results from **temporal expectations** with regard to the onset of distracting events – although there is to date very little empirical work on this topic. In that work, **temporal structure** is tied to the predictability of the inter-stimulus-interval or to the periodic appearance of a stimulus (Balke et al., 2021; Lamy, 2005; Nobre & van Ede, 2018; Xu et al., 2021). Whether proactive suppression that results from temporal expectations occurs at an absolute-feature, dimensional-salience, or overall-salience level remains undetermined (→ ***Features***).

Numerous EEG studies have investigated proactive distractor inhibition using spectral analysis of oscillatory power. Unlike event-related potentials, such as the Pd or N2pc (see below), which represent evoked responses following a stimulus event, oscillatory power can be observed before such events, making it a promising tool for studying proactive distractor suppression before stimulus onset. Specifically, strong alpha power reflects a state of relatively greater physiological inhibition and is often interpreted as reflecting attenuated sensory processing (Jensen & Mazaheri, 2010; Samaha et al., 2020). Accordingly, spatially informative cues in a wide variety of attention tasks typically induce lateralization of alpha-band power such that power is relatively reduced, indicating disinhibition, over the hemisphere processing relevant information (de Vries et al., 2019; Foxe & Snyder, 2011). In search tasks, cueing the location of the upcoming *target* (Worden et al., 2000) or statistical learning of the most likely target location (Noonan et al., 2016) yields robust alpha-band lateralization towards the target, that is, reduced power contralateral to the target, in the interval before array onset. Although the mechanisms involved in cueing and statistical learning are likely to be different, the presence of lateralized alpha to cued and statistically learned targets suggests that lateralized alpha reflects the allocation of spatial attention towards likely target locations. In line with this, one study found that target predictability strengthened the typical effect of target cueing on lateralized alpha power, suggesting that attention and prediction may interact in biasing sensory processing (Alilović et al., 2019). In contrast, evidence for equivalent alpha lateralization by cueing (Foster & Awh, 2019) or learning *distractor* locations has been mixed. While several studies have found stronger alpha power in the hemisphere contralateral to an expected distractor (van Zoest et al., 2021; Wang et al., 2019; Zhao et al., 2023), roughly the same number of studies using similar paradigms have failed to find such effects (Ferrante et al., 2023; Noonan et al., 2016; van Moorselaar & Slagter, 2019, 2020). Thus, the relationship between the mechanisms underlying changes in alpha power to proactive target selection and those involved in proactive distractor inhibition are still debated (Foster & Awh, 2019; Noonan et al., 2018).

Other findings are more difficult to classify as indexing proactive suppression relative to the distractor’s appearance versus stimulus-triggered suppression. In fMRI, the imprecise timing of BOLD precludes clear inferences from the finding that BOLD visual responses to expected salient distractors are attenuated beginning in V1 (Adam & Serences, 2021; Won et al., 2020; B. Zhang et al., 2021). Likewise, the findings that the proportion of first saccadic eye movements to a salient distractor falls below that of non-salient distractors (Gaspelin et al., 2017) or that oculomotor capture decreases when salient distractors appear in high- versus low probability distractor locations (Di Caro et al., 2019; Sauter et al., 2021; Wang et al., 2019) might reflect suppressive processes that occur at either time point.

**Stimulus-triggered suppression** may operate through synaptic changes that only modulate stimulus processing when a postsynaptic response is triggered (Briggs et al., 2013). These latent/silent/quiescent changes (Stokes, 2015) in response to expected distractors can be more difficult to measure using common methods in psychological science, such as EEG oscillations, ERPs, or fMRI BOLD because these have spatial and temporal resolutions that are orders of magnitude coarser than the synaptic scale. Nevertheless, changes at the synaptic level can be inferred through clever indirect measures (Duncan et al., 2022). In electrophysiological studies, the P_D_ ERP component (Hickey et al., 2009) is often taken to indicate the presence of stimulus-triggered suppression when it occurs around 100–200 ms (see Cosman et al., 2018, for non-human primate electrophysiology; Gaspelin & Luck, 2018b; Sawaki & Luck, 2010; Stilwell et al., 2022, for human ERPs). This early P_D_ is present before the first shift of attention and can occur without a direct shift of attention, as indexed by the N2pc beginning around 200 ms (Chelazzi et al., 1993; Luck & Hillyard, 1994).

**Reactive** suppression following attentional capture can be inferred when distractor suppression follows a measurable shift of attention to the critical distractor. For example, the P_D_ ERP component has been taken to indicate reactive suppression when it occurs after an N2pc component to the distractor (Liesefeld et al., 2017, 2022; van Moorselaar & Slagter, 2019). Interestingly, reactive suppression as indexed by the late P_D_ was found to decrease for expected distractors (van Moorselaar et al., 2020; van Moorselaar & Slagter, 2019), suggesting that distractor suppression may also come about through learning-based mechanisms not indexed by the P_D_. With eye tracking, reactive suppression is often measured as shorter fixation dwell times on a distractor that has captured the first saccade after stimulus onset compared to a neutral distractor (Geng & Diquattro, 2010; Sauter et al., 2021; Theeuwes, 2010; Z. Zhang et al., 2022).

To summarize, disagreements about what should constitute a reference event for defining what is proactive versus reactive suppression has led to some confusion in distraction research. Here, we suggest it is important to clearly distinguish between the three putative timings of suppression that are currently debated in the literature by specifying the reference event when using the terms *proactive* and *reactive* suppression. In addition, we suggest using the term *stimulus-triggered suppression* whenever one wants to specifically refer to suppression that occurs after the distractor appears and before it captures attention). Future research may develop more precise terms based on newly identified mechanisms that operate at different time scales and in response to different stimulus triggers. At that time, the new terms should be adopted to reflect developments in the field.

### Part 2: Glossary

#### Abrupt onset

A stimulus that is presented suddenly at a location previously unoccupied by any other stimulus and phenomenologically “appears out of nowhere.”

#### Absolute feature

A feature that a stimulus possesses irrespective of other stimuli in the scene. Examples are a specific color, shape, or orientation. This contrasts with properties that are defined only in relation to features of other stimuli, such as salience or singleton status (i.e., relative features). → ***Features***

#### Attentional capture

An involuntary shift of attention towards a stimulus that is not a target (i.e., a distractor or non-target → ***Stimuli***). This presumably happens when the priority (→ ***Guidance***) of the distractor is highest in the visual field. → ***Types of distraction***

#### Attentional control setting/Attentional set

A preparatory state of the information processing system that prioritizes specific information for selection and further processing. Attentional control settings include the *target template* as well as other task-relevant information. → ***Templates and strategies***

#### Attentional guidance

Any factor that modulates relative activation levels on a *priority map* and thereby contributes to determine what location or stimulus in the visual field wins the competition for attention at a given time, is said to guide attention. → ***Guidance***

#### Attentional priority

The probability that a stimulus in the visual field will receive attention relative to other stimuli. It is dynamically determined by the combination of a variety of influences that have different time courses. In the context of a priority map, *attentional priority* refers to the relative strength of activation at each location of the map. → ***Priority map***

#### Attentional strategy

A mental plan, or policy, guiding how attentional control is used in an attempt to satisfy task goals. Strategies can sometimes lead to establishing a specific *target template*, but they can also consist of more abstract policies, such as “search for the most valuable target,” “search through the less numerous subset of stimuli,” or “withhold saccadic eye movements until the target is located covertly.” → ***Templates and strategies***

#### Attentional suppression

Any mechanism by which the attentional processing of a stimulus/location/time is reduced relative to a specific reference point. This could be at the level of an *absolute feature* (first-order suppression), at the level of salience within a feature dimension (second-order suppression) or at a global salience level (→ ***Features***). A**ttentional enhancement** refers to any mechanism by which the attentional processing of a stimulus/location/time is increased. → ***Modulation***

#### Bottom-up guidance/stimulus-driven guidance

Refers to characteristics of the stimulus display that influence *attentional guidance*. See also *salience map.* → ***Guidance***

#### Contingent capture effect

A behavioral or neural effect resulting from attention being more robustly captured by distractors, the more these match the observer’s *search-guiding features.* → ***Common paradigms***

#### Cue

This term is used in two very different ways. (1) Informative cue: A stimulus, typically presented prior to the search display, that carries task-relevant information (e.g., the target’s likely location, feature, or onset time). (2) Non-informative cue. A *distractor* that typically does not occur at the same time as the search display. → ***Stimuli***

*Recommendation.* Make sure to use either “**informative cue**” or “**non-informative cue”**.

#### Dimension weighting

A principle whereby salience signals for each feature dimension are weighted before integration on an overall priority map. Dimensional weights are thought to be affected by goal-directed guidance and selection history. → ***Templates and strategies***

#### Distraction

Processing of irrelevant information that impairs search performance. *Attentional capture* and *filtering costs* are specific instances of distraction. → ***Types of distraction***

#### Distractors and nontargets

In the broader visual-search literature all irrelevant stimuli are relatively interchangeably referred to as nontargets or distractors. → ***Stimuli***

#### Recommendation

Reserve the word *distractor* to refer to the stimuli for which the potential to cause distraction is examined. When necessary, specify what type of distractor is meant by adding an attribute such as **(target-feature) matching distractor** or **singleton distractor** in order to clearly demarcate it from other irrelevant stimuli in the respective task. Reserve the term **nontargets** to refer to irrelevant stimuli that are not distractors.

#### Feature-search mode

An *attentional strategy* in which a specific feature-based *target template* (e.g. red) is implemented, in contrast to salience-based search in *singleton-detection mode.* Not to be mistaken with the term “feature search”, which describes a search paradigm in which the target is defined as unique in at least one feature dimension (e.g., Treisman & Gelade, 1980). → ***Templates and strategies***

#### Filtering costs

A delay in responding to a target caused by processing simultaneously present, distractors in the absence of *attentional capture* by these distractors. → ***Types of distraction***

#### Goal-directed attention

The intentional prioritization of locations, features or moments in time that are construed by the observers as relevant in order to perform the task at hand. → ***Guidance***

#### Negative template

A representation of features known not to characterize the target and that may be used to deprioritize/reject/suppress/ignore a subset of distractors. Also sometimes referred to as *template for rejection*. → ***Templates and strategies***

#### Optimal tuning of attention

Adjustments of the *target template* that reduce the overlap between the search-guiding feature and features of irrelevant stimuli and thereby increase the signal-to-noise ratio. → ***Templates and strategies***

#### Paradigm

In the study of distraction, a paradigm refers to an experimental procedure that is characterized by a core manipulation and a core comparison of interest, although its variants may differ in many respects. The main paradigms used to study distraction in visual search are the additional-singleton, irrelevant-singleton, spatial-cueing, dot-probe, letter-probe, spatial-blink and distractor-feature cueing paradigms. Some less frequently used paradigms combine diverse elements from different paradigms. → ***Paradigms***

#### Pre-attentive feature

A feature of a stimulus that can be processed before attention is allocated towards that stimulus. The mental representation of a stimulus’ features might change when that stimulus is focally attended relative to when it is not. → ***Features***

#### Priority map

A spatial representation that codes *attentional priority* at each location and is blind to the sources of that prioritization.

*Recommendation:* Terms like *activation map* and *conspicuity map* could have uses in specific theoretical settings but, ideally, would not be used as synonyms of *salience map* or *priority map*.

#### Proactive modulation

A task-related change in pre-stimulus baseline activity or in the latent/silent/quiescent representation of target or distractor features that affects processing of upcoming visual stimuli. In the context of visual search, this results in the enhancement or suppression of stimulus representations. → ***Timing***

*Recommendation:* To avoid confusion, it is important to define the reference event that triggers the proactive process (i.e., stimulus onset, first shift of attention). We also suggest using the term *stimulus-triggered modulation* when specifically referring to a change that occurs after the critical event (e.g., the target or distractor) and before the first shift of attention. Note that proactive and reactive modulations are not mutually exclusive in that both can theoretically be applied to the same stimulus on the same trial.

#### Reactive modulation

A task-related change in the processing of ongoing visual information following a reference event. In the context of visual search, this reference event is often the first shift of attention or the search-display onset. → ***Timing***

*Recommendation:* To avoid confusion, it is important to define the reference event that triggers the reactive process (i.e., stimulus onset, first shift of attention). We also suggest using the term *stimulus-triggered modulation* when specifically referring to a change that occurs after the critical event (e.g., the target or distractor) and before the first shift of attention. Note that proactive and reactive modulations are not mutually exclusive in that both can theoretically be applied to the same stimulus on the same trial.

#### Relational coding

A principle whereby stimuli are represented relative to the other stimuli in the display and not in terms of their *absolute features*. Thus, the same relational coding may be obtained with different absolute features. → ***Templates and strategies***

#### Reported feature

The feature of a stimulus that determines the response. In some paradigms it is crucial that this feature cannot be detected pre-attentively, because it might otherwise act as the *search-guiding feature*. → ***Features***

#### Salience

The local feature contrast of a stimulus in one or multiple feature dimensions. Salience is a continuous feature and it is therefore unclear at which level of salience a stimulus should be considered “salient” (as opposed to “non-salient”). However, claiming that stimulus A is more salient than Stimulus B is often less controversial, in particular if both stimuli are salient in the same feature dimension. → ***Features***

#### Salience map

The spatial representation of the visual distinctiveness of a feature, stimulus, or location (see *salience*), driven by feedforward, stimulus-driven (or bottom-up) processes. → ***Priority map***

*Recommendation: Priority map* and *salience map* are sometimes used interchangeably, but we encourage the use of salience map only when referring exclusively to stimulus-driven factors.

#### Scene guidance

Modulation of the priority map by aspects of the scene/display that are independent of the presence or features of the target. → ***Guidance***

#### Search-guiding feature

The feature that is used for guiding attention towards the target. By definition, this feature must be processed pre-attentively. → ***Features***

#### Search performance

In most of the paradigms used to study distraction in visual search, search performance refers to the speed or accuracy at performing the search task. → ***Tasks***

#### Selection history

Prior experience, broadly construed, that exerts a direct influence on *attentional guidance.* Current usage often includes all sources of attentional guidance other than goals and salience. The most studied selection history phenomena include *inter-trial priming*, *statistical learning*, *learned associations* between stimuli and outcomes (i.e., reward/punishment learning). Such guidance is typically considered to be driven by implicit mechanisms. → ***Guidance***

#### Set size

The number of stimuli in a search display. → ***Stimuli***

#### Singleton

A stimulus that has a (locally) unique feature in any feature dimension. For example, the unique red stimulus among green stimuli is a color singleton. In contrast to *salience* which is continuous, a stimulus either is a singleton or is not. → ***Features***

#### Singleton-detection mode

An *attentional strategy* that is wholly salience driven and is thus optimized for detecting salient singleton stimuli, in contrast to *feature search mode*. Also referred to as *discrepancy detection mode* or simply *salience-driven search*. → ***Templates and strategies***

#### Suppression effect

A behavioral or neural effect indicating that a stimulus’ attentional priority is *below* baseline level. Researchers have to establish a well-reasoned baseline and then compare processing of the stimulus in question to this baseline. A baseline could be some type of stimulus, experimental condition, or time period. An **enhancement effect** is a behavioral or neural effect indicating that a stimulus’ attentional priority is *above* a pre-defined baseline. → ***Modulation***

*Recommendation:* It is important to distinguish suppression and enhancement effects from their underlying mechanisms. Also, only use *suppression effect* to mean a reduction in measured attentional priority relative to a well-defined baseline.

#### Suppression/inhibition

The action of preventing, hindering, or interrupting production of another action or thought that would otherwise have happened. In psychology, the term refers to a voluntary or involuntary restraint that prevents or hinders the direct expression of an impulse, cognitive process, or action. In the neural / computational domain, it refers to a mechanism whereby one component of a network prevents or reduces activation of another component. → ***Modulation***

*Recommendation: Suppression* and *inhibition* are often used synonymously. Make sure to follow the naming conventions of your area (e.g., *inhibition of return*) and make sure to specify exactly what you mean while using these terms.

#### Target

A stimulus observers are instructed to find within a search display. Instructions define targets either by one feature (“Look for a circle”), by multiple features (“Look for a red circle”), by a category (“Look for the animal”) or by stating in which feature dimension they stand out (“Look for a shape singleton”). All other stimuli in the search display are referred to as **irrelevant stimuli**. → ***Stimuli***

#### Target-defining feature

The feature of a stimulus that defines it as the target according to task instructions. It may also be used as a *search-guiding feature*, but not necessarily. → ***Features***

#### Target template

A core concept in models of attention that refers to the representation of target information held in working or long-term memory during visual search. A target template is thought to be used to set attentional priority (*search-guiding features*), and/or determine whether or not a stimulus is the target (*target-defining features*). It is interchangeably referred to as the *attentional template*, *mental template*, or *search template* (see also *attentional control setting*). → ***Templates and strategies***

*Recommendation:* It is essential to distinguish the target template that contains the complete representation of the target in memory from information about the target that is used for guidance and/or information used for decisions about target identity (see *search-guiding feature* and *target-defining feature)*.

#### Task

A task refers to what participants are informed to do (e.g., “Search for a given letter and report its color”). Tasks used to study distraction are *detection* (“Is the target present or absent?”), *localization* (“Where is the target?”) and, most often, *discrimination* (*compound*/*classification*; “Which *reported feature* does the target possess?”). Nevertheless, the term *task* is often used to refer to a paradigm (e.g., *letter-probe task*). → ***Tasks***

#### Temporal expectation

The implicit or explicit utilization of temporal structure in service of *attentional guidance*. → ***Timing***

#### Temporal structure

Information that can be used to prioritize/deprioritize moments in time. Examples include: cued associations, hazard rates, rhythms and sequences. → ***Timing***

#### Top-down guidance

Researchers refer to top-down guidance in two different ways. (1) For some, in keeping with the definition of top-down processes in perception, top-down guidance refers to any type of guidance that is not stimulus driven, and therefore includes both goal-directed attention and selection history. (2) For others, top-down guidance is synonymous with *goal-directed attention*. → ***Guidance***

*Recommendation:* If using the term *top-down*, specify what definition is adopted.

## Appendix: Genesis

We believe that the present project can have value beyond research on just visual distraction. It can provide a model for future attempts to standardize terminology in other areas of psychology and neuroscience. To facilitate such future attempts, we here provide a detailed account of the steps that produced this paper. This is not meant as a prescription on how to approach similar projects, but merely as a source of inspiration on how such projects might work out.

**Funding and administrative support**

This project was sparked by a generous grant from the Ludwig-Maximilians-Universität’s Center for Advanced Studies (CAS^LMU^) awarded to Hermann J. Müller and Heinrich R. Liesefeld. This funding of the Research Group *Handling Visual Distraction* allowed assembling many of the world-leading experts on the topic. The original main purpose of this grant was to fund extended research visits (several months) of a few selected researchers in Munich to work on, or develop, projects together with the grant recipients on a dedicated topic. The proposal was written in 2019, the funding decision was communicated in August of 2020 with an official start date in October 2021. Due to the COVID-19 pandemic the original plan was not feasible. Instead of spending the money on a few long visits, we decided to concentrate all visits into a short period at the end of the funding period in July and August 2022, when we would also have a conference on visual distraction. We hoped that international travel would be possible again. It turned out that the structure of several months of online meetings followed by an extended physical co-presence of the group members in one place, which emerged out of the constraints imposed by COVID-19, was highly productive: It yielded many interesting discussions, papers and other projects on the topic. Most importantly, it shifted the overall focus from a few bilateral collaborations to a real community endeavor. We are indebted beyond measure to the friendly and helpful CAS^LMU^ staff for enduring all the stress that this co-opting and overstraining of the generous resources must have meant to them.

**Getting started**

Leading up to the actual research visits, we had several online meetings. In a kick-off meeting (15 October 2021), the group decided to work on specific topics in several sub-groups, as pitched by group members at the meeting. The group on “Terminology” that gave rise to the present paper had a dedicated kick-off meeting to discuss the approach and create a first list of terms that we would need to define. Some of the group members had already participated in similar attempts to arrive at a consensus with regard to theory (Luck et al., 2021) and methodology (Wöstmann et al., 2022), and their experiences were valuable to avoid various pitfalls, including overly optimistic expectations on the degree of consensus, potential for long-winded and tedious discussions on minor points, and proper acknowledgement of intellectual contributions. We therefore decided to try to define terms in a way that is compatible with many theoretical views, to allow for various definitions of the same term, to provide the opportunity for individual authors to embrace specific definitions (which was not needed in the end), and to keep track of individual contributions.

**Composition of the group**

It was clear from the beginning that a project on terminology cannot be done in private, so we wanted to include as much of the community as possible. The downside of including too many people, of course, is that with too many opinions and too many busy schedules to manage, the process can become prohibitively slow, effectively preventing the project from happening. As a compromise, we wrote this article with only a share of the community – a selection that was necessarily artificial to a certain degree: some of the group members were directly invited by the speakers of the CAS Research Group and some answered to a call on *visionlist* and in *Nature* with an application that was selected by the speakers. This two-step procedure ensured a good balance of senior and junior scientists and that all major theoretical standpoints were represented. At some point we had also considered asking the community to vote on their preferred definition (if multiple definitions existed for the respective term) via online tools and via a voting booth at the annual meeting of the *Vision Science Society.* However, it turned out that we had a sufficiently large consensus and that multiple definitions were needed for only a handful of terms, for which it seemed more reasonable to provide several definitions without any weighting.

**Structuring the process and writing the article**

After we had produced a first version of the introduction, this appendix, and a few definitions, we created a table listing to-be-defined terms (that was extended by additional terms throughout the whole process as appeared sensible), in which all interested members of the group could indicate their interest in writing or revising each individual definition. This table also provided a coarse structure by suggesting clusters of terms and a sensible order; these clusters were initially sorted into broader categories, namely, *stimuli*, *paradigms*, *mental representations*, *cognitive mechanisms*, *outcomes and measures*, which might also apply to many other topics in cognitive science. Despite providing an overarching structure during an early phase of the project, these categories turned out to hamper the writing in some places and were therefore eventually dropped. By contrast, clusters of terms were maintained and had two important functions: (a) some of the terms are so strongly related that it was most appropriate to discuss them in relation to each other, both during group discussions and in the final paper; (b) this allowed distributing the work and parallelizing the process by forming subgroups (see below).

In the next phase, we wrote definitions and prose and commented on the developing texts in parallel in a single online document. After a while, the online document became too chaotic with the comments far exceeding the actual text in volume. At this point, we formed subgroups for each cluster. In particular, HRL suggested subgroup compositions and lead authors for each subgroup; this was based on expressed interest (in the aforementioned table or otherwise) and/or participation in the preceding discussions and work on the online document. Depending on their interests, participants were allowed to sign in and out of as many subgroups as they liked at any time and also scale their involvement (from actively writing text to reading and commenting on existing text). Either or both of the two first authors (DL and HRL) participated in (almost) each of the groups most of the time and thereby assured communication across subgroups.

At first, it was unclear whether we wanted the final paper to contain dictionary-style definitions or essays. An online survey did not help us decide on that matter, because the group was almost evenly split on the question. Rethinking the issue revealed that both styles have advantages and are actually complementary. We therefore decided to include both a glossary and essays.

This is the stage at which we met each other in person. After a 4-day conference at lake Ammersee near Munich, which we used to update each other on our respective lines of work, most authors of the present paper stayed in Munich for days or weeks to continue the work on this and other projects. We scheduled meetings of the whole group, as well as meetings of subgroups working on their respective cluster of terms. In a general group discussion, we discovered that some of the subgroups had started working on the same terms, despite our efforts to closely coordinate the work and the fact that many participants contributed to several groups. To avoid further redundancy, we decided to first produce the definitions for all terms in each subgroup, before writing the prose. This also proved useful for establishing a maximally broad consensus.

Accordingly, each subgroup was asked to first come up with a set of definitions that achieved consensus within the subgroup, during one or several subgroup meetings. Based on the premise that any definition must be based on a particular set of theoretical, often unverified, assumptions, we agreed to provide definitions that are general enough to be valid across theoretical standpoints. Thus, we agreed to formulate definitions at a level on which the theoretical overlap is sufficiently large. Finally, we decided to allow entries listing conflicting definitions, instead of finding vacuous compromises. Each subgroup was required to add their definitions to an online document until a set deadline. Second, everybody (i.e., authors across all subgroups) was encouraged to read through those definitions and flag definitions for which they thought further discussion or changes were required until another deadline a few days later. Third, the whole group met to discuss the flagged definitions to resolve remaining issues; these discussions took several hours – much longer than we had expected (see Hofstadter, 1999, p. 152) and, thus, required a second meeting.

Once we had agreed upon the definitions, the lead authors wrote or revised their respective essays and had the other subgroup members comment on them serially (sending a text document via email) until a final version that all subgroup members agreed upon was created. This final version was then pasted into an online document accessible to the whole group.

All the produced material was structured, formatted and revised by the lead author in consultation with some of the other group members, resulting in a first draft of the paper that was then opened for discussion by the whole group. At this point, we also decided on the author byline, based on the amount of effort and ideas each of the group members had contributed. In particular, the two first authors had clearly contributed most, followed by the subgroup leads. Thus, subgroup leads are listed before all remaining contributors and within these categories the order is alphabetical.

**Final reflection**

The present project has cost a lot of time and effort from a large group of busy and successful scientists. Given this huge investment, one might consider projects of this type as inefficient. However, the present paper is not the only outcome of our discussions. Notably, most participants found the process itself highly rewarding, not least because these discussions have tremendously increased our knowledge of the literature and our mutual understanding of alternative theoretical positions. Looking back at this arduous effort and forward to how much more could be done in both depth and scope, what we have achieved feels like a first tiny step though not lacking flaws. Nevertheless, we believe and hope that this paper will catalyze scientific progress and so be of high value for the visual-distraction community and beyond. As the ancient Chinese saying goes: “A journey of a thousand miles begins with a single step.” We hope that we have placed this step well and that the wanderer will keep going – and get some company!

## Footnotes

1 We thank Clayton Hickey for contributing this valuable insight to our project.

2 For a more in-depth and apt treatment of these topics than we could provide here, please refer to the philosophical works of Ludwig Wittgenstein and Jacques Derrida, for example.
